# The prevalence and factors associated with mental disorders in a community setting in central Uganda

**DOI:** 10.1371/journal.pone.0285091

**Published:** 2023-05-04

**Authors:** Dickens Akena, Ronald Kiguba, Wilson W. Muwhezi, Brendan Kwesiga, Gwendolyne Kigozi, Hafsa Lukwata, Noeline Nakasujja

**Affiliations:** 1 Department of Psychiatry, Makerere University College of Health Sciences, Kampala, Uganda; 2 Department of Pharmacology, Makerere University College of Health Sciences, Kampala, Uganda; 3 Health Systems Strengthening Cluster, World Health Organization, Kenya Country Office, Gigiri, Kenya; 4 Grants office, Makerere University College of Health Sciences, Kampala, Uganda; 5 Department of Mental Health, Ministry of Health of Uganda, Kampala, Uganda; The Hong Kong Polytechnic University, HONG KONG

## Abstract

**Background:**

Mental disorders are known to predict poverty, morbidity and mortality. In resource limited settings, low levels of mental health literacy (MHL) and high mental illness stigma (MIS) have been sighted as possible factors that may impede access to mental health care. However, little has been done to examine the association between mental disorders and these factors (MHL and MIS) in sub-Saharan Africa.

**Methods:**

We assessed for the prevalence of major depressive disorders (MDD), substance use disorders (SUD), post-traumatic stress disorder (PTSD), generalized anxiety disorder (GAD), documented MHL and MIS among 814 participants from 24 villages in central Uganda. We conducted regression analyses to examine the association between the prevalence of mental disorders, demographic factors as well as MIS and MHL.

**Results:**

Over two thirds of the participants 581 (70%) were female. The mean age of the participants was 38 years (SD± 13.5). The prevalence of mental disorders ranged from 6.8–32%. Participants who were older were less likely to screen positive for GAD (OR 0.98; 0.96–0.99), female gender was protective against SUD (OR 0.46; 0.3–0.68) and those with MDD had lower education level (OR 0.23; 0.1–0.53). The mean MIS score was 11.3 (SD± 5.4) with a range of 6–30 and the mean MHL score was 21.7 (SD ±3.0) with a range of 10–30. MIS was negatively associated with GAD [β = -1.211 (-2.382 to -0.040)]. There no statistically significant association between MHL and a mental disorder.

**Conclusion:**

There was a high prevalence of mental disorders in the community that we studied. Adequate resources should be allocated to address this burden.

## Background

A number of studies including those conducted in low resourced sub-Saharan Africa (SSA) settings have reported a high prevalence of common mental disorders including major depressive disorders (MDD) at 4.5%, generalized anxiety disorder (GAD) at 3.5%, post-traumatic stress disorders (PTSD) at 8% and substance use disorders (SUD) at 1.3% [1–5]. Mental disorders are known to independently predict poor outcomes including but not limited to increased mortality [6–8], poverty [9] and morbidity [10–13].

A number of scholars have recommended that mental disorders are routinely screened for in primary health care settings and treated using psychological therapies (mild to moderate cases) or pharmacotherapy (moderate to severe cases) [14–17]. However, literature shows that there are a number of factors that may hinder adequate identification and treatment of common mental disorders. These factors are worth considering when planning for the integration of mental health care services in SSA.

Health care system barriers such as shortage of mental health providers [18, 19] medication stock-outs [20, 21] and poor funding for mental health [22, 23] have been cited as reasons for the poor access to mental health care services. These barriers generally lead to poor detection of mental illnesses and low uptake of interventions. In a bid to reduce the treatment gap for mental disorders, the World Health Organization devised the mental health gap action program in 2010. Whereas the WHO program was adapted in multiple SSA countries [24–26], a number of individual barriers continue to peg back moves towards the integration of mental health care. These barriers (see below) are worth mentioning.

High levels of mental illness stigma (MIS) [27, 28] and low levels of mental health literacy (MHL) [29, 30] have been documented in SSA. MHL is defined as the ability of an individual to recognize mental illnesses, know about their causes and seek help from both lay and professional sources Jorm (1997) [31]. Individuals with low MHL may not be aware that they are experiencing mental health symptoms for which there are medical remedies. Moreover, individuals who harbor stigma towards mental illness may not report symptoms to health care providers even if they are aware that they are suffering from a mental illness. Similarly, community members who harbor mental illness stigma may be reluctant to refer mentally ill patients to health care facilities or deny them basic services because of their condition—despite the availability of mental health care services within lower level health care (health centers III and IV) facilities found at every parish in the community. Referral of patients for mental health care is likely to happen when patients are grossly disruptive as is the case for people with acute psychotic disorders or bipolar affective disorders in manic phases. Despite the presence of MIS and MHL in SSA, little has been done to examine its association with common mental disorders.

Furthermore, a number of studies have shown that some individuals in SSA harbor non-medical explanatory models of mental illnesses—such persons attribute their ailments to spiritual possession or some evil forces [32, 33] and not a medical illness for which there is treatment. Such individuals may not seek treatment at health facilities. These non-medical explanatory models of mental illness can also be a result of low MHL or high MIS levels.

Studies that examine the association between MIS, MHL and the prevalence of common mental disorders are likely to help in shaping policy and practice regarding the integration of mental health care in primary health care settings in low resourced SSA. Our study objective was to document the prevalence of common mental disorders and examine whether they were associated with MHL and MIS in a community sample of individuals in central Uganda.

## Methods

### (i) Study design

This cross sectional study was conducted in 24 randomly chosen villages in the districts of Kampala, Mukono, Masaka and Wakiso in the period of November to December 2020. A village is the lowest politico-administrative unit often consisting of between 50 to 70 households and home to anywhere between 250 to 1,000 people [34].

### (ii) Sample size calculation

We used the Leslie Kish formular [35] for calculating the sample size for cross sectional studies. The formula states that: n = Z^2^ p (1-p)/E^2.^ Where n- sample size, E-The standard error (5%), p- Proportion of patients; 50%. The prevalence was estimated at 50% as the conservative estimates for outcomes or effects sizes with limited literature to refer to (in this case, prevalence of mental disorders among community members in Uganda). Z- The standard normal deviation of 1.96 corresponding to 95% confidence interval. Substituting; n = 1.96^2^ X 0.5 X (1–0.5)/0.05^2^ n = 384. We used a community correction effect of 2. Thus, the sample size was determined to be 764. We added 10% to the computed sample size to cater for incomplete data mainly due to failure to complete an interview. Thus, the total sample size was 840 participants

### (iii) Eligibility criteria

Individuals were eligible for recruitment if they had been residing in the selected villages for a minimum of 6 months, and were at least 18 years old. All participants were required to provide written informed consent. For individuals who could neither read nor write, the consent form was read to them by a research assistant. Such participants used a thumb print soaked in an ink-pad to provide informed consent. Consent forms and study instruments were translated into Luganda, the local language dialect largely spoken in the study districts.

### (iv) Translation

First, we sent the English versions of the consent and study questionnaires for direct translation into the Luganda language by persons who spoke both languages. We then provided the translated versions to mental health specialists who were native Luganda speakers who assessed the instruments for clarity of language; they provided some input and the documents were sent back to the translators to modify the text as recommended. The Luganda version was sent to an independent translator to have it back translated into English; the final text was then compared and a consensus between the forward and back translators in the presence of the mental health experts was reached.

### (v) Sampling

We got a list of all the villages in the four districts of Kampala, Mukono, Wakiso and Masaka from the Uganda National Bureau of Statistics website [36]. The four districts have a population of approximately 5,785,600 persons, which is ~12.6% of the total population of Uganda (45,741,000). We then selected the villages using the probability proportional to size approach based on number of villages per district. Thus, our sample consisted of 15 out of 3297 villages from Kampala district, 4 out of 813 villages from Mukono district, 2 out of 436 villages from Masaka district and 3 out of 704 villages from Wakiso.

We employed a multistage sampling approach to select 840 participants from 24 randomly selected villages located in the 362 parishes (5,250 villages) that span the study districts.

### (vi) Participant identification

We engaged the village community leaders to help in identification of every 12^th^ household in their community till they accrued 38 participants in each village. This figure was based on an average of 4,710 households per village—thus 4,710/38 which approximately translated into every 12^th^ household.

### (vii) Study measures

We used the following instruments during our data collection. All instruments were translated into Luganda. Data collection (November-December 2020) was done by trained research assistants. Research assistants physically administered the questionnaires to each participant in a face to face interview.

- A standardized demographic questionnaire to collect age, gender, education level and place of residence information.
- We used the Mental health Knowledge Schedule (MAKS) [37] to document MHL and MIS. The MAKS comprises 6 stigma-related mental health knowledge areas including help-seeking, recognition, support, employment, treatment, and recovery and 6 items which inquire about knowledge of mental illness conditions including depression, generalized anxiety, substance use disorders, stress, schizophrenia and bipolar disorders. It is scored 1–5 with the first six items determining stigma and the last 6 determining mental health literacy. The MAKS has not been validated for use in Uganda (we reported on the chronbac alpha)
- The Generalized Anxiety Disorder scale (GAD-7) [38] was used to assess for GAD. We used a cut off score of 10, which is indicative of moderate to severe generalized anxiety disorder. A cut off score of ≥10 yields a sensitivity of 89% and a specificity of 82% for GAD.
- The Patient Health Questionnaire-9 (PHQ-9) [39] was used to assess for MDD. The PHQ-9 is a Nine-item instrument based on the Diagnostic and Statistical Manual (DSM). The scale assesses all the 9 DSM symptoms of MDD and is scored 0–3 with higher scores indicative of a possible depression. A cut off score of ≥10 is used to denote possible depression.
- The Post-Traumatic Stress Disorder- Primary Care Screen (PTSD-PC) [40] was used to assess for PTSD. The PTSC-PC is a 5 item instrument with a cut-point of 3 being optimally sensitive to probable PTSD.
- The Alcohol Smoking and Substance Involvement screening Test (ASSIST) [41] was used to assess for SUD. The ASSIST was developed by the World Health Organization to as tool for the early identification of substance use related health risks and substance use disorders in primary health care, general medical care and other settings. It screens for hazardous, harmful and dependent use of 10 substances including alcohol, tobacco, cannabis, cocaine, opioids, amphetamines, inhalants, sedatives, Hallucinogens and other psychoactive drugs. A high scores indicates a likelihood of hazardous use.

### (vii) Data analysis

Descriptive statistics were used to present demographic data. The prevalence of MDD, GAD, PTSD and SUD (dependent variables) was calculated using the cut-off scores (above) and reported as percentages. We conducted simple linear regression analyses to determine the factors that were associated with the outcomes of interest and reported β-coefficients, odds ratios and confidence intervals. The independent variables were demographic parameters (age, gender, education level) and address. Variables that showed statistical significance at the bivariate level were then entered into a hierarchical regression model (one), controlling for age and level of education as potential confounders. The level of significance was set to P < .05. All of the statistical analyses were performed using STATA software version 14.

### Ethics approval and consent to participate

Ethical approval was obtained from Makerere University School of Medicine Research Ethics Committee (SOMREC reference number 2020–176), and Uganda National Council for Science and Technology (reference number HS1009ES). All participants provided written informed consent. The study was conducted as per guidelines set by the relevant approving entities above. All research assistants were trained to respond appropriately by providing counseling, terminating the interview or contacting the PI for participants who are severely distressed and need urgent care.

## Results

We recruited 814 participants, 29% (n = 233) of whom were male. The mean age of the participants was 38 years (SD± 13.5). Only 60% of the participants had attained at least a secondary education.

The prevalence of screen positive cases for a mental disorders ranged from 6–32% (Table 1). There was a high comorbidity between alcohol use disorders and PTSD as well as GAD and depression disorder (Table 1). Individuals who screened positive for depression were more likely to have low levels of education, OR 0.23 (0.1–0.53), p <0.001. Participants who were older were less likely to screen positive for GAD OR 0.98 (0.96–0.99), p = 0.017 while female gender was protective against the use of alcohol OR 0.46 (0.3–0.68) p<0.001 (Table 2). There was no statistically significant association between geographical location and the prevalence of the mental disorders and the associated factors of MIS and MHL.

**Table 1:** Demographic and clinical characteristics.

Demographic characteristics	Frequency (%)
Age in years, mean (SD)	38 (13.5)
**Age categorized, n (%)**	
18–30 years	287 (35)
31–49 years	366 (45)
50–91 years	161 (20)
**Sex, n (%)**	
Male	233 (29)
Female	581 (71)
**Education level, n (%)**	
None	43 (5)
Primary	284 (35)
Secondary	357 (44)
Tertiary	130 (16)
**Clinical Parameters**	
Stigma, mean (SD)	11.3 (5.4)
Mental Health Literacy, mean (SD)	21.7 (3.0)
PHQ-9 > 9, n (%)	56 (6.8%)
GAD-7 > 10, n (%)	93 (11%)
PTSD-sc > 5, n (%)	263 (32%)
Alcohol, n (%)	125 (15.3%)
**Comorbidities of the Mental Disorders**	
PTSD and Alcohol, n = 156	35 (22.8%)
PTSD and Depression, n = 156	17 (10.8%)
Depression (PHQ-9 > 10) and Alcohol, n = 49	10 (20%)
Depression (PHQ-9 > 10) and Generalized Anxiety (GAD-7> 10), n = 49	25 (51%)

**Table 2:** Multivariable logistic regression analyses of demographic factors associated with PTSD, depression, GAD, alcohol use disorders.

	PTSD*		Depression^±^		GAD^±^		Alcohol Use Disorder~	
	OR (95% CI)	p-value	OR (95% CI)	p-value	OR (95% CI)	p-value	OR (95% CI)	p-value
**Sex**								
Male	1.00		1.00		1.00		1.00	
Female	0.84 (0.61, 1.16)	0.290	0.88 (0.46, 1.69)	0.699	1.20 (0.73, 1.99)	0.474	0.46 (0.30, 0.68)	**<0.001**
**Age**	0.99 (0.98, 1.01)	0.354	1.02 (0.99, 1.04)	0.150	0.98 (0.96, 0.99)	**0.017**	0.99 (0.98, 1.01)	0.439
**Education**								
None	1.00		1.00		1.00		1.00	
1^0^ to 3^0^	0.73 (0.38, 1.40)	0.344	0.23 (0.10, 0.53)	**0.001**	0.55 (0.23, 1.31)	0.178	0.84 (0.34, 2.09)	0.711

*Score >4;

^±^Moderate to severe disease (score>10);

~Moderate to high risk for alcohol use disorder 1^0^ to 3^0^ Primary-tertiary education

The mean MIS score was 11.3 (SD± 5.4) with a range of 6–30 and the mean MHL score was 21.7(SD ±3.0) with a range of 10–30. The chronbac alpha for the MIS was 0.73 (item variance of 0.59) and that of the MHL was 0.69 (item variance of 0.58). There was no statistically significant association between MIS and demographic parameters including age, gender and geographical location. However, there was a negative association between MIS and the presence of a GAD (p = 0.043), Table 3. We found statistically significant association between MHL and education level (p = 0.003) but not with a mental disorder or geographical location (Table 4).

**Table 3:** Bivariate and multivariable analysis for MIS ***predictor variable being MIS.

Demographic and outcome variables	n (%)	Unadjusted β-coefficient	95% CI	p-value	Adjusted β-coefficient	95% CI	p-value
**Age in years**	814 (100)	-0.017	-0.044–0.011	0.236	-0.019	-0.047–0.009	0.183
**Gender, n (%)**							
Female (vs. Male)	581 (71)	-0.568	-1.389–0.254	0.175	-0.547	-1.372–0.278	0.193
**Education**							
1^0^ to 3^0^ (vs. None)	284 (35)	0.462	-1.199–2.124	0.585	0.115	-1.573–1.803	0.893
**Mental Disorders**							
GAD-7 > 10, n (%)	93 (11)	-1.175	-2.340 - -0.009	0.048	-1.211	-2.382 - -0.040	0.043*

**Table 4:** Bivariate and multivariable analysis for MHL ***predictor variable being MHL.

Demographic and outcome variables	n (%)	Unadjusted β-coefficient	95% CI	p-value	Adjusted β-coefficient	95% CI	p-value
**Age in years**	814 (100)	0.005	-0.011–0.20	0.557	0.008	-0.007–0.024	0.302
**Gender, n (%)**							
Female (vs Male)	581 (71)	-0.056	-0.513–0.402	0.811	0.019	-0.439–0.477	0.936
**Education**							
1^0^ to 3^0^ (vs. None)	284 (35)	1.319	0.400–2.239	0.005	1.378	0.461–2.334	**0.003** *

*1^0^ to 3^0^ = Primary to tertiary education

## Discussion

We report findings from one of the first studies to document the prevalence of mental disorders, MHL and MIS as factors that may impede access to care in a community sample of persons in a low resourced setting.

**The prevalence of mental disorders** has been shown to vary widely in SSA. For example, the prevalence of PTSD has been shown to range from 4% to 25% in the general population and up to 30% in community samples of persons affected by conflict [4, 42]. Our finding of a 32% prevalence of PTSD is much higher than what has been documented in non-conflict community settings in a meta-analysis by Ng et al (2020) [4]. One possible explanation to the high prevalence documented in our study could be as a result of a surge in exposure to violence during a civil unrest that was witnessed in the country in mid to late 2020—there were multiple reports of high handed responses by security forces against members of the public leading to general elections.

The prevalence of MDD in our study (6.8%) was lower than findings from a community survey that was conducted in Kenya (prevalence 15.6%) [43] as well as those from a meta-analysis of findings from Africa (11.5%) [44–46]. The reasons for the low prevalence of MDD in our study will need further examination. We documented an 11% prevalence of GAD that was equally lower than the 15.7% that was reported in the community survey in Kenya [43], but way higher than figures from the world mental health surveys and global burden of disease findings [47]. The explanation for our findings could be a result in differences in population dynamics as well as survey measures used in data collection. Just like in the case of MDD, the reasons for the low prevalence of GAD will need further scrutiny

We reported a high prevalence of AUD in the study population. Our findings are in keeping with previous studies that have documented high prevalence of consumption of alcohol in Uganda [48–50] with figures as high as 40% in one study [51]. These findings are in keeping with those of a meta-analysis of the prevalence of AUD (40%) in SSA by Olawole et al (2018) [52]. A number of factors including the indiscriminate advertising of alcohol as well as weak laws that are unable to curb production of alcoholic beverages may explain the high prevalence of alcoholic beverage use in Uganda.

The prevalence of PTSD in our study was way higher than findings from a community survey that was conducted in Northern Uganda [53] and almost as high as those reported in conflict areas and refugee populations in the country [54, 55]. We already alluded to a surge in election related violence in some communities in the country in mid to late 2020—this could explain the high prevalence of PTSD in the study population and is in keeping with findings from a meta-analysis that shows a much higher prevalence in conflict areas of SSA [4].

### Factors associated with mental disorders

At multi-variable logistic regression, we found that young age was associated with GAD, female gender was protective against AUD and low education was associated with depression. Our findings regarding the GAD have been reported in studies [56, 57] especially during pandemics. A possible reason could be that the younger people were more worried and uncertain about what the future held for them especially at a time when the world was undergoing the COVID-19 pandemic—the pandemic caused significant disruption in academic calendars and job opportunities for the younger generation leading to feelings of despair and uncertainties about the future.

We documented a negative association between AUD and female gender. Studies in SSA have documented low prevalence of AUD among females compared to male [51, 58]. In our situation, the association between a low prevalence of AUD and females gender could be explained by the nature of, and uniqueness of the study setting. In Uganda, like other SSA settings, the consumption of alcohol may not fully embraced, let alone culturally sanctioned among females compared to males. Our findings about the low education being a predictor of depression has been documented in multiple studies [59–62]. We believe that the same factors (higher socioeconomic status among the educated, being more embedded in socially supportive structures, personality characteristics) at play explain this association.

### MIS and MHL

Our findings showed high MHL and moderate MIS levels. The high MHL findings could be an indication of progress made by the ministry of health (MOH) of Uganda that set out to deliberately raise awareness about the burden of mental illnesses in communities, especially after the onset of the COVID-19 pandemic. An explanation for the moderate levels of MIS could be a result of deeply ingrained belief systems and explanatory models about the etiology of mental illnesses in the population [32, 33]. Our findings also indicate that persons with low levels of education are likely to have low scores on the MHL. A number of studies have reported low education as being a predictor of poor MHL [63, 64]—our results are in keeping with these findings. We were not able to demonstrate an association between MHL and the presence of mental disorders. The reasons for this finding could be due to the relatively high levels of MHL and low prevalence of mental disorders in the community that we studied. The reverse association between GAD and MIS requires further scrutiny.

### Recommendations

Our findings indicate a prevalence of mental disorders. Efforts have to be put in place to combat mental illness among members of the population.

### Limitations

One of the limitations of this study is that it was cross sectional and thus we are not able to determine any causative factors.

## Supporting information

S1 File (XLSX)

## References

[1] Jörns-Presentati Astrid, Napp Ann-Kathrin, Dessauvagie Anja S., Stein Dan J., Jonker Deborah, Breet Elsie, et al. The prevalence of mental health problems in sub-Saharan adolescents: A systematic review. PLoS ONE 2021;16(5):e0251689. 10.1371/journal.pone. 33989357 PMC8121357

[2] Gouda Hebe N, Charlson Fiona, Sorsdahl Katherine, Ahmadzada Sanam, Ferrari Alize J, Erskine Holly, et al. Burden of non-communicable diseases in sub-Saharan Africa, 1990–2017: results from the Global Burden of Disease Study 2017. Lancet Glob Health. 2019;7(10):e1375–e87. doi: 10.1016/S2214-109X(19)30374-2 31537368

[3] GBD 2019 Mental Disorders Collaborators*. Global, regional, and national burden of 12 mental disorders in 204 countries and territories, 1990–2019: a systematic analysis for the Global Burden of Disease Study 2019. Lancet Psychiatry. 2022:S2215-0366(21)00395-3. 10.1016/S2215-0366(21)00395-3 35026139 PMC8776563

[4] Ng Lauren C., Stevenson Anne, Kalapurakkel Sreeja S., Charlotte HanlonI, Soraya Seedat, Boniface Harerimana, et al. National and regional prevalence of posttraumatic stress disorder in sub-Saharan Africa: A systematic review and meta-analysis. PLOS Medicine 2020;17(5): 10.1371/journal.pmed.1003090 PMC7228043 32413027

[5] GBD 2016 Alcohol and Drug Use Collaborators. The global burden of disease attributable to alcohol and drug use in 195 countries and territories, 1990–2016: a systematic analysis for the Global Burden of Disease Study 2016. The Lancet Psychiatry. 2018;5(12):987–1012 DOI:10.6/S2215-0366(18)30337-7. doi: 10.6/S2215-0366(18)30337-7 30392731 PMC6251968

[6] Elizabeth Reisinger Walker, Robin E. McGee, Benjamin G. Druss. Mortality in Mental Disorders and Global Disease Burden Implications; A Systematic Review and Meta-analysis. JAMA Psychiatry. 2015;72(4):334–41. doi: 10.1001/jamapsychiatry.2014.2502 25671328 PMC4461039

[7] Too Lay San, Spittal Matthew J, Bugeja Lyndal, Reifels Lennart, Butterworth Peter, Pirkis Jane. The association between mental disorders and suicide: A systematic review and meta-analysis of record linkage studies. Journal of Affective Disorders. 2019;259:302–13. doi: 10.1016/j.jad.2019.08.054 31450139

[8] Correll Christoph U., Solmi Marco, Veronese Nicola, Bortolato Beatrice, Rosson Stella, Santonastaso Paolo, et al. Prevalence, incidence and mortality from cardiovascular disease in patients with pooled and specific severe mental illness: a large-scale meta-analysis of 3,211,768 patients and 113,383,368 controls. World Psychiatry. 2017;16:163–80. doi: 10.1002/wps.20420 28498599 PMC5428179

[9] Ridley Matthew, Rao Gautam, Schilbach Frank, Patel Vikram. Poverty, depression, and anxiety: Causal evidence and mechanisms. Science. 2020;11(6522):eaay0214. doi: 10.1126/science.aay0214 33303583

[10] Whiteford Harvey A., Ferrari Alize J., Degenhardt Louisa, Feigin Valery, Vos Theo. The Global Burden of Mental, Neurological and Substance Use Disorders: An Analysis from the Global Burden of Disease Study 2010. Plos One. 2015;10(2):e0116820. doi: 10.1371/journal.pone.0116820 25658103 PMC4320057

[11] Vigo Daniel V, Kestel Devora, Pendakur Prof Krishna, Thornicroft Prof Graham, Atun Prof Rifat. Disease burden and government spending on mental, neurological, and substance use disorders, and self-harm: cross-sectional, ecological study of health system response in the Americas. The Lancet Public Health. 2019;4(2):E89–E96. doi: 10.1016/S2468-2667(18)30203-2 30446416

[12] Almeida Osvaldo P., Hankey Graeme J., Yeap Bu B., Golledge Jonathan, Norman Paul E., Flicker L. Mortality among People with Severe Mental Disorders Who Reach Old Age: A Longitudinal Study of a Community-Representative Sample of 37892 Men. PLoS ONE 2014;9(10):e111882. 10.1371/journal.pone.0111882. 25360781 PMC4216120

[13] Charlson Fiona J., Diminic Sandra, Lund Crick, Degenhardt Louisa, Whiteford Harvey A.. Mental and Substance Use Disorders in Sub-Saharan Africa: Predictions of Epidemiological Changes and Mental Health Workforce Requirements for the Next 40 Years. PLoS ONE 2014;9(10):e110208. 10.1371/journal.pone.0110208. 25310010 PMC4195720

[14] Lopes Alessandra Pereira, Macedo Tânia Fagundes, Coutinho Evandro Silva Freire, Figueira Ivan, Ventura Paula Rui. Systematic Review of the Efficacy of Cognitive-Behavior Therapy Related Treatments for Victims of Natural Disasters: A Worldwide Problem. Plos One. 2014;9(10):e109013. 10.1371/journal.pone.0109013. 25296020 PMC4189911

[15] Brown R. C., Witt A., Fegert J. M., Keller F., Rassenhofer M., Plener P. L.. Psychosocial interventions for children and adolescents after man-made and natural disasters: a meta-analysis and systematic review. Psychological Medicine 2017;47:1893–905. doi: 10.1017/S0033291717000496 28397633

[16] Hoskins Mathew, Pearce Jennifer, Bethell Andrew, Dankova Liliya, Barbui Corrado, Tol Wietse A., et al. Pharmacotherapy for post-traumatic stress disorder: systematic review and meta-analysis. The British Journal of Psychiatry. 2015;206:93–100. doi: 10.1192/bjp.bp.114.148551 25644881

[17] Sullivan Gregory M, Neria Yuval. Pharmacotherapy in post-traumatic stress disorder: Evidence from randomized controlled trials. Curr Opin Investig Drugs. 2009;10(1): 35–45. 19127485 PMC3630071

[18] Burvill P.W.. Looking beyond the 1:10,000 Ratio of Psychiatrists to Population Australian & New Zealand Journal of Psychiatry. 2017;26(2):265–9. 1642618

[19] Psychiatrists and nurses (per 100 000 population) [Internet]. 2017 [cited 09.August. 2017].

[20] Wagenaar Bradley H., Stergachis Andy, Rao Deepa, Hoek Roxanne, Cumbe Vasco, Manuel Napúa, et al. The availability of essential medicines for mental healthcare in Sofala, Mozambique. Global Health Action 2015;8(1):10.3402/gha.v8.27942. 26081970 PMC4469619

[21] Docrat Sumaiyah, Besada Donela, Cleary Susan, Daviaud Emmanuelle, Lund Crick. Mental health system costs, resources and constraints in South Africa: a national survey Health Policy and Planning. 2019;34(9):706–19, 10.1093/heapol/czz085. 31544948 PMC6880339

[22] Molodynski Andrew, Cusack Christina, Nixon Jurua. Mental healthcare in Uganda: desperate challenges but real opportunities. BJPsych Int 2017;14(4):98–100. doi: 10.1192/s2056474000002129 29093962 PMC5663025

[23] Raja Shoba, Wood Sarah K, Victoria de Menil, Mannarath SC. Mapping mental health finances in Ghana, Uganda, Sri Lanka, India and Lao PDR. International Journal of Mental Health Systems. 2010;4(11): doi: 10.1186/1752-4458-4-11 20507558 PMC2889860

[24] Jessica Spagnolo, Shalini Lal. Implementation and use of the Mental Health Gap Action Programme Intervention Guide (mhGAP-IG): A review of the grey literature. J Glob Health. 2021;(PMC8053394):doi: 10.7189/jogh.11.04022. 10.7189/jogh.11.04022 PMC8053394 33884192

[25] Faregh Neda, Lencucha Raphael, Ventevogel Peter, Benyam Worku Dubale, Kirmayer Laurence J.. Considering culture, context and community in mhGAP implementation and training: challenges and recommendations from the field. International Journal of Mental Health Systems. 2019;3(58):10.1186/s13033-019-0312-9. 31462908 PMC6708207

[26] Keynejad Roxanne C, Dua Tarun, Barbui Corrado, Thornicroft Graham. WHO Mental Health Gap Action Programme (mhGAP) Intervention Guide: a systematic review of evidence from low and middle-income countries. Evid Based Mental Health. 2017:doi:10.1136/eb-2017-102750. PMC10283403 28903977

[27] Egbe Catherine O, Brooke-Sumner Carrie, Kathree Tasneem, Selohilwe One, Thornicroft Graham, Petersen Inge. Psychiatric stigma and discrimination in South Africa: perspectives from key stakeholders. BMC Psychiatry. 2014;14(191):10.1186/471-244X-14-191. 24996420 PMC4099203

[28] Kapungwe A, Cooper S, Mwanza J, Mwape L, Sikwese A, Kakuma R, et al. 2010. Afr J Psychiatry (Johannesbg). Mental illness—stigma and discrimination in Zambia.;13(3):192–203. 20957318

[29] Ganasen K.A, Parker S, Hugo C.J, Stein D.J, Emsley R.A, Seedat S. Mental health literacy: focus on developing countries. Afr J Psychiatry (Johannesbg). 2008;11(1):23–8. doi: 10.4314/ajpsy.v11i1.30251 19582321

[30] Atilola Olayinka. Level of community mental health literacy in sub-Saharan Africa: Current studies are limited in number, scope, spread, and cognizance of cultural nuances Nordic Journal of Psychiatry 2015;69(2):93–101. 25151995 10.3109/08039488.2014.947319

[31] Jorm A.F. Mental Health Literacy. Public Belief about Mental Disorders. British Journal of Psychiatry. 2000;177:396–01. 10.1192/bjp.177.5.396 11059991

[32] Makanjuola Victor, Esan Yomi, Oladeji Bibilola, Kola Lola, Appiah-Poku John, Haris Benajamin, et al. EXPLANATORY MODEL OF PSYCHOSIS: IMPACT ON PERCEPTION OF SELF STIGMA BY PATIENTS IN THREE SUB-SAHARAN AFRICAN CITIES. Soc Psychiatry Psychiatr Epidemiol. 2016;51(12):1645–54, doi: 10.007/s00127-016-1274-8. doi: 10.1007/s00127-016-1274-8 27491966 PMC6311698

[33] Cohen Alex, Padmavati Ramachandran, Hibben Maia, Oyewusi Samuel, John Sujit, Esan Oluyomi, et al. Concepts of madness in diverse settings: a qualitative study from the INTREPID project. BMC Psychiatry 2016;388(16):10.1186/s12888-016-1090-4. doi: 10.1186/s12888-016-1090-4 27829384 PMC5103598

[34] Richard Kavuma. Explainer: Local government structures in Uganda: The Guardian; 2010 [cited 2021 28.09.2021]. https://www.theguardian.com/katine/2009/dec/14/local-government-explainer].

[35] Kish Leslie. An edition of Survey sampling. New York: J. Wiley; 1965. 627–35 p.

[36] Uganda Bureau of Statistics. https://www.ubos.org/explore-statistics/20/ 2020.

[37] Evans-Lacko Sara, Little Kirsty, Meltzer Howard, Rose Diana, Rhydderch Danielle, Henderson Claire, et al. Development and Psychometric Properties of the Mental Health Knowledge Schedule. Can J Psychiatry. 2010;55(7):440–8. 20704771 10.1177/070674371005500707

[38] Robert L Spitzer, Kurt Kroenke, Williams Janet B. W., Löwe Bernd. A Brief Measure for Assessing Generalized Anxiety Disorder: The GAD-7. Arch Intern Med. 2006;166(10):1092–7. doi: 10.1001/archinte.166.10.1092 16717171

[39] Kroenke K, Spitzer R.L, Williams J.B. The PHQ-9: validity of a brief depression severity measure. Journal of General Internal Medicine,. 2001;16(9):606–13 doi: 10.1046/j.1525-1497.2001.016009606.x . 11556941 PMC1495268

[40] Prins Annabel, Bovin Michelle J., Smolenski Derek J., Marx Brian P., Kimerling Rachel, Jenkins-Guarnieri Michael A, et al. The Primary Care PTSD Screen for DSM-5 (PC-PTSD-5): Development and Evaluation Within a Veteran Primary Care Sample. J Gen Intern Med 2016;31(10):1206–11. doi: 10.1007/s11606-016-3703-5 27170304 PMC5023594

[41] The Alcohol, Smoking and Substance Involvement Screening Test (ASSIST): manual for use in primary care. [Internet]. World Health Organization 2010.

[42] Sekoni Olutoyin, Mall Sumaya, Prevalence Christofides N. and factors associated with PTSD among female urban slum dwellers in Ibadan, Nigeria: a cross-sectional study. BMC Public Health volume. 2021;21 (1546):10.1186/s12889-021-1508-y. PMC8359091 34384401

[43] Kwobah Edith, Epstein Steve, Mwangi Ann, Litzelman Debra, Atwoli Lukoye. Prevalence of psychiatric morbidity in a community sample in Western Kenya. BMC Psychiatry 2017;17(30):10.1186/s12888-017-1202-9. PMC5242046 28100210

[44] Lim Grace Y., Tam Wilson W., Lu Yanxia, Ho Cyrus S., Zhang Melvyn W., Ho RC. Prevalence of Depression in the Community from 30 Countries between 1994 and 2014. Nature, Scientific Reports. 2018;8(2861):10.1038/s41598-018-21243-x. 29434331 PMC5809481

[45] Ayano G, Solomon M, Abraha M. A systematic review and meta-analysis of epidemiology of depression in people living with HIV in east Africa. BMC Psychiatry. 2018;18(1):254. Epub 2018/08/17. doi: 10.1186/s12888-018-1835-3 ; PubMed Central PMCID: PMC6094569. 30111300 PMC6094569

[46] Bernard Charlotte, Franc¸ois Dabis, Nathalie de Rekeneire. Prevalence and factors associated with depression in people living with HIV in subSaharan Africa: A systematic review and metaanalysis. PLoS ONE 2017;12(8):e0181960. 10.1371/journal.pone. doi: 10.1371/journal.pone 28783739 PMC5544236

[47] Baxter Amanda J., Vos Theo, Scott Kate M., Norman Rosana E., Flaxman Abraham D., Blore Jed, et al. The regional distribution of anxiety disorders: implications for the Global Burden of Disease Study, 2010. Int J Methods Psychiatr Res. 2014;23(4):422–38. doi: 10.1002/mpr.1444 25048296 PMC6878273

[48] Nalwadda Oliva, Rathod Sujit D., Nakku Juliet, Lund Crick, Prince Martin, Kigozi Fred. Alcohol use in a rural district in Uganda: findings from community-based and facility-based cross-sectional studies. International Journal of Mental Health Systems,. 2018 12(12):10.1186/s13033-018-0191-5. PMC5883606 29632551

[49] Kabwama Steven Ndugwa, Ndyanabangi Sheila, Mutungi Gerald, Wesonga Ronald, Bahendeka Silver K., Guwatudde David. Alcohol use among adults in Uganda: findings from the countrywide non-communicable diseases risk factor cross-sectional survey. Glob Health Action. 2016;9:10.3402/gha.v9.31302. doi: 10.3402/gha.v9.31302 27491961 PMC4974493

[50] Kamulegeya Louis Henry, Kitonsa Peter James, Okolimong Eric, Kaudha Gloria, Maria Sonia, Nakimuli-Mpungu Etheldreda. Prevalence and associated factors of alcohol use patterns among university students in Uganda. Pan African Medical Journal. 2020;37:339. doi: 10.11604/pamj.2020.37.339.21136 33738027 PMC7934195

[51] Wagman Jennifer A., Nabukalu Dorean, Miller Amanda P., Wawer Maria J., Ssekubugu Robert, Nakowooya Hadijja, et al. Prevalence and correlates of men’s and women’s alcohol use in agrarian, trading and fishing communities in Rakai, Uganda. Plos One. 2020;15(10):e0240796. 10.1371/journal.pone. doi: 10.1371/journal.pone 33125397 PMC7598464

[52] Olawole-Isaac A., OgundipeII O., O AmooI E., Adeloye D. Substance use among adolescents in sub-Saharan Africa: A systematic review and meta-analysis. S Afr j child health 2018;12:10.7196/sajch.2018.v12i2.1524

[53] Mugisha James, Muyinda Herbert, Wandiembe Peter, Eugene Kinyanda Prevalence and factors associated with Posttraumatic Stress Disorder seven years after the conflict in three districts in northern Uganda (The Wayo-Nero Study). BMC Psychiatry. 2015;15(170). 10.1186/s12888-015-0551-5 PMC4513792 26205192

[54] Winkler Nina, Ruf-Leuschner Martina, Verena Ertl, Pfeiffer Anett, Schalinski Inga, Ovuga Emilio, et al. From war to classroom: PTSD and depression in formerly abducted youth in Uganda. Front Psychiatry,. 2015;6(2):10.3389/fpsyt.2015.00002. 25788887 PMC4348469

[55] Achille Mwira Bapolisi, Song Suzan J, Kesande Claire, Godfrey Zari Rukundo, Scholastic Ashaba. Post-traumatic stress disorder, psychiatric comorbidities and associated factors among refugees in Nakivale camp in southwestern Uganda. BMC Psychiatry. 2020;20(53):10.1186/s12888-020-2480-1. PMC7006164 32033558

[56] Varma Prerna, Junge Moira, Meaklim Hailey, Jackson Melinda L.. Younger people are more vulnerable to stress, anxiety and depression during COVID-19 pandemic: A global cross-sectional survey. Progress in Neuropsychopharmacol Biol Psychiatry. 2021;13:110236. doi: 10.1016/j.pnpbp.2020.110236 33373680 PMC7834119

[57] Gambin Małgorzata, Marcin Sękowski Małgorzata Woźniak-Prus, Wnuk Anna, Oleksy Tomasz, Cudo Andrzej, et al. Generalized anxiety and depressive symptoms in various age groups during the COVID-19 lockdown in Poland. Specific predictors and differences in symptoms severity. Comprehensive Psychiatry. 2021;105:152222. doi: 10.1016/j.comppsych.2020.152222 33388494

[58] Martinez Priscilla, Røislien Jo, Nirmala Naidoo, Clausen Thomas. Alcohol abstinence and drinking among African women: data from the World Health Surveys. BMC Public Health 2011;11(160): doi: 10.1186/1471-2458-11-160 21392398 PMC3061917

[59] Kessler Ronald C., Bromet Evelyn J.. The Epidemiology of Depression Across Cultures. Annual Review of Public Health. 2013;34:119–38. doi: 10.1146/annurev-publhealth-031912-114409 23514317 PMC4100461

[60] Kate Josje ten, Koster Willem de, Jvd Waal. Why are Depressive Symptoms More Prevalent Among The Less Educated? The Relevance of Low Cultural Capital and Cultural Entitlement. Sociological Spectrum,. 2017;37(2):63–76

[61] Peyrot W.J, Lee S.H, Milaneschi Y, Abdellaoui A, Byrne E.M, Esko T, et al. The association between lower educational attainment and depression owing to shared genetic effects? Results in ~25,000 subjects. Mol Psychiatry. 2015;6:735–43. doi: 10.1038/mp.2015.50 25917368 PMC4610719

[62] McFarland Michael J., Brandon G. Wagner. Does a College Education Reduce Depressive Symptoms in American Young Adults? Soc Sci Med 2015;146:75–84. 26513116 10.1016/j.socscimed.2015.09.029 PMC4676078

[63] Kaneko Y, Motohashi Y. Male Gender and Low Education with Poor Mental Health Literacy: A Population-based Study. Journal of epidemiology / Japan Epidemiological Association. 2007;17:114–9. doi: 10.2188/jea.17.114 17641446 PMC7058472

[64] Furnham A, Swami V. Mental Health Literacy: A Review of What It Is and Why It Matters. International Perspectives in Psychology: Research, Practice, Consultation. 2018;7. doi: 10.1037/ipp0000094.

